# Complete Genome Sequence of Achromobacter spanius UQ283, a Soilborne Isolate Exhibiting Plant Growth-Promoting Properties

**DOI:** 10.1128/MRA.00236-19

**Published:** 2019-04-18

**Authors:** Taylor J. Wass, Sharifah Farhana Syed-Ab-Rahman, Lilia C. Carvalhais, Brett J. Ferguson, Peer M. Schenk

**Affiliations:** a Algae Biotechnology Laboratory, School of Agriculture and Food Sciences, The University of Queensland, Brisbane, Queensland, Australia; b Plant-Microbe Interactions Laboratory, School of Agriculture and Food Sciences, The University of Queensland, Brisbane, Queensland, Australia; c Queensland Alliance for Agriculture and Food Innovation, The University of Queensland, Brisbane, Queensland, Australia; d Centre for Integrative Legume Research, School of Agriculture and Food Sciences, The University of Queensland, Brisbane, Queensland, Australia; University of Maryland School of Medicine

## Abstract

Achromobacter spanius UQ283 is a soilborne bacterium found to exhibit plant growth-promoting and disease-suppressing attributes in several plant species. Accordingly, we used long-read sequencing to determine its complete genome sequence. The assembled genome will aid in understanding the multifaceted interactions between plant growth-promoting rhizobacteria, pathogens, and plants.

## Abstract (precis)

Achromobacter spanius UQ283 is a soilborne bacterium found to exhibit plant growth-promoting and disease-suppressing attributes in several plant species. Accordingly, we used long-read sequencing to determine its complete genome sequence.

## ANNOUNCEMENT

Achromobacter spanius UQ283 is a Gram-negative, rod-shaped bacterium isolated from the Arabidopsis thaliana rhizosphere. Members of this species have been identified in various samples, including those from freshwater and soil, and in human clinical isolates, and the type strain, *A. spanius* CCUG 47062, originated from the blood of a cystic fibrosis patient (1, 2). The genome of *A. spanius* UQ283 was of interest because preliminary screening showed that it exhibited attributes of plant growth-promoting rhizobacteria, such as phosphate solubilization, indole-3-acetic acid biosynthesis, siderophore production, and *in planta* antioomycete activity against Phytophthora cinnamomi (3).

*A. spanius* UQ283 was isolated from the rhizosphere of between 8 and 12 leaf-stage *A. thaliana* ecotype Col-0 plants grown in University of California soil mix amended with a compost soil extract (1 ml per seedling). This extract was prepared by mixing compost soil (Green Fingers B2 potting mix, Nerang, Australia) into autoclaved water to a final concentration of 3.3% (wt/vol). *A. spanius* UQ283 was isolated from a rhizosphere soil suspension (1% [wt/vol]) inoculated on National Botanical Research Institute phosphate growth medium (NBRIP) agar and stored as 20% glycerol stocks at −80°C for further experiments (4).

We obtained the complete genome sequence of *A. spanius* UQ283 using single-molecule real-time sequencing (SMRT; Pacific Biosciences, CA). A volume of 50 µl of glycerol stock was used to inoculate 15 ml of lysogeny broth (LB) and incubated overnight at 28°C (5). Cells were harvested with centrifugation, and we removed the supernatant and resuspended it in 1.5 ml of LB. DNA was extracted from this sample with the Qiagen Gentra Puregene Cell kit per the manufacturer’s instructions. The resulting DNA was submitted to the Ramaciotti Institute (Sydney, Australia) for sequencing on a PacBio RS II system, using one SMRT cell with P6-C4 chemistry and a 240-minute movie length. We constructed 20-kb SMRTbell libraries according to the manufacturer’s protocols and size selected them (15 to 50 kb) using the BluePippin electrophoresis system (Sage Science, Beverly, MA, USA). Sequencing yielded 902 Mb of data from 88,598 reads after subread filtering, with a mean length of 10.2 kb and *N*_50_ value of 14,782 bp. Assembly was performed with the Hierarchical Genome Assembly Process (v3) as part of the SMRT analysis environment (v1.87.139483). Default parameters were used for all software unless otherwise noted. Polishing with Quiver achieved a consensus accuracy greater than 99.9985% (QV 48), and the assembly was circularized with Circlator (v1.5.3) (6). The final assembly consisted of one 6,687,826-bp contig, with a GC content of 63.81%, representing the *A. spanius* UQ283 nuclear genome at an average coverage of 109×. Genome annotation was performed with the NCBI Prokaryotic Genome Annotation Pipeline, which identified 5,914 protein coding sequences, 17 rRNA genes, and 60 tRNA genes (7). Average nucleotide identity based on BLAST (ANIb) matching to members of *Achromobacter* was computed using JSpeciesWS (v3.1.2), with *A. spanius* CGMCC9173 (98.18%), *A. spanius* DSM 23806 (92.07%), and Achromobacter piechaudii (86.13%) returning the highest identity values (8).

### Data availability.

The assembly and annotation were deposited in GenBank under accession number CP034689. The BioProject accession number is PRJNA511771, and the Sequence Read Archive accession number is SRR8607288.
